# Prediction of culture-positive sepsis and selection of empiric antibiotics in critically ill patients with complicated intra-abdominal infections: a retrospective study

**DOI:** 10.1007/s00068-020-01535-6

**Published:** 2020-11-03

**Authors:** Joohyun Sim, Sung Soo Hong, Jae Young Kwak, Yun Tae Jung

**Affiliations:** 1 Department of Surgery, Ajou University School of Medicine, Suwon, Republic of Korea; 2 Department of Surgery, National Medical Center, Seoul, Republic of Korea; 3 Department of Surgery, Gangneung Asan Hospital, University of Ulsan College of Medicine, Gangneung, Republic of Korea

**Keywords:** Antibiotic, Critically ill, Intra-abdominal infection

## Abstract

**Purpose:**

To compare the mortality rates between culture-positive and culture-negative sepsis in complicated intra-abdominal infections (cIAI) and investigate the predictors of culture-positivity and their causative microorganisms.

**Materials and methods:**

The medical records of 1581 adult patients who underwent emergency gastrointestinal surgery between January 2013 and December 2018 were reviewed retrospectively. A total of 239 patients with sepsis or septic shock who were admitted to an emergency department, underwent emergency surgery for cIAI, and needed postoperative intensive care unit care were included and divided into two groups according to their initial blood and peritoneal culture results.

**Results:**

Among the 239 patients, 200 were culture-negative and 39 were culture-positive. The culture-positive group had higher in-hospital (35.9% vs 14.5%; *P* = .001) and 30-day mortality (30.8% vs 12.0%; *P* = .003) than the culture-negative group. Colon involvement (OR 4.211; 95% CI 1.909–9.287; *P* < .001) and higher Sequential Organ Failure Assessment (SOFA) score (OR 1.169; 95% CI 1.065–1.282; *P* = .001) were shown to be the predictors of culture-positive sepsis for cIAI. Regarding antibiotic sensitivity, 31.6% of the gram-positive bacteria were methicillin-resistant and 42.1% of the gram-negative bacteria were extended spectrum β-lactamase-producing Enterobacteriaceae.

**Conclusions:**

Patients with cIAI had higher mortality rates in culture-positive sepsis than in culture-negative sepsis. High SOFA score and colon involvement were the risk factors associated with culture-positivity. The most common single species grown in the blood or peritoneal cultures was *Escherichia coli*, and the most common group was Gram-positive cocci.

**Electronic supplementary material:**

The online version of this article (10.1007/s00068-020-01535-6) contains supplementary material, which is available to authorized users.

## Introduction

Sepsis is one of the most common causes of mortality in hospitalized patients [1, 2]. From the early 1990s to the middle of 2010s, the definition of sepsis was generally accepted as having systemic inflammatory response with suspected source of infection [3]. Recently, a third definition of sepsis, Sepsis-3, reestablished sepsis as life-threatening organ dysfunction caused by a dysregulated host response to infection, with organ dysfunction represented by an increase in the Sequential Organ Failure Assessment (SOFA) score of 2 points or more [4]. The organ dysfunction is caused either by the infection of the affected organ itself or by the inflammatory responses to infection such as flush of inflammatory cytokines, increased vascular permeability, and decreased intravascular volume leading to less tissue oxygenation [5].

Identification of the sepsis origin and the causative microorganisms followed by appropriate selection of empirical antibiotics and prompt source control is critical for its treatment [6, 7]. According to previous studies, culture studies failed to prove any source of infection in a large number of patients who are suspicious of having sepsis. Approximately 30–89% of patients with sepsis were reported to have negative culture results [8–14]. Several hypotheses have been developed to explain the low yield of detecting microorganisms in these septic patients. These hypotheses include prior antibiotic treatment, insufficient sampling of blood, transport problems, and insufficient technique [9].

Nannan Panday et al. [9] reported that culture-positive sepsis was associated with higher 28- and 90-day mortalities and involvement of multiple organ systems dysfunction. However, there are debates on whether culture-positive sepsis is associated with higher rates of mortality and adverse outcomes [8, 9, 15–19]. Sigakis et al. [8] showed that patients with culture-negative and culture-positive sepsis demonstrated similar characteristics as well as similar mortality after adjusting for severity of illness.

There are not many studies comparing the mortality rates between the culture-positive and culture-negative patient groups with complicated intra-abdominal infections (cIAI). Therefore, the primary aim of this study to evaluate the association between culture results and the mortality rates in these patients. The secondary aims were to investigate the predictors for culture-positive sepsis in cIAI and their causative microorganisms with their resistance to antibiotics.

## Methods

### Study design

**Fig. 1:**
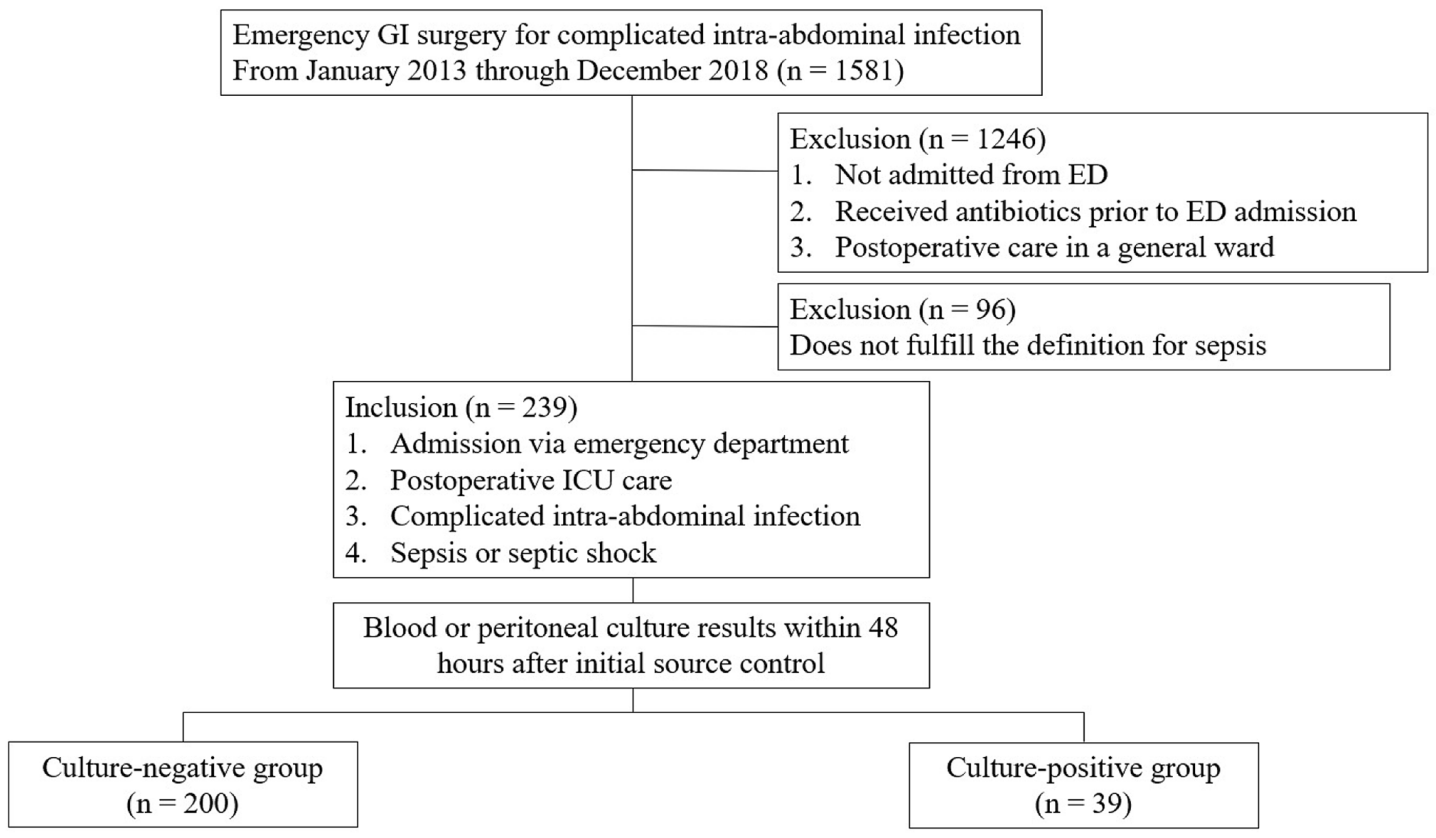
Study population. *GI* gastrointestinal; *ED* emergency department; *ICU* intensive care unit

Electronic medical records of 1581 patients who underwent emergency gastrointestinal (GI) surgery for intra-abdominal infection from January 2013 to December 2018 in a tertiary medical center were reviewed. 239 patients with sepsis or septic shock were included for analysis with following inclusion criteria: patients who admitted via emergency department (ED), received no antibiotics prior to the ED admission, required postoperative intensive care unit (ICU) care, and underwent surgery for cIAI. These patients were classified into culture-negative and culture-positive groups according to the initial blood or peritoneal culture results (Fig. 1).

### Data collection and definition

Sepsis is defined as organ dysfunction caused by infection represented by increased SOFA score of 2 or more from the baseline. Septic shock is defined as sepsis with vasopressor required to maintain a mean arterial pressure greater or equal to 65 mmHg and a serum lactate level greater than 2 mmol/L [4]. In this study, sufficient information needed to calculate SOFA score could be obtained after the patients arrived at ICU. Therefore, “Septic shock” in Table 2 reflects the condition of the patients in the immediate postoperative period. IV hydrocortisone was used for patients with refractory septic shock when hemodynamic stability had not been achieved after adequate fluid resuscitation and vasopressor therapy.

Antibiotics were chosen based on the guideline published from the World Society of Emergency Surgery [20]. A combination of third-generation cephalosporin and metronidazole was the most common antibiotic regimen administered to the study population because patients who had recent history of hospitalization were excluded. Antibiotics were used at ED as the patients were suspected to have cIAI.

“Culture positive” was confirmed when one or more microorganisms were identified in initial blood or peritoneal cultures. Two sets of blood cultures were routinely obtained at ED for patients who were suspected for sepsis or septic shock. Specimens for peritoneal cultures were collected immediately after surgery in the ICU from Jackson-Pratt drainage bags. The ejection holes of the bags were sterilized by povidone-iodine balls before collecting fluids aseptically from the silicone suction reservoirs. Sensitivity data of identified microorganisms to various antibiotics were also collected. “Culture negative” was assumed when common contaminants such as coagulase-negative staphylococci, Bacillus species, and micrococci were identified in only one blood cultures.

Data regarding baseline characteristics for the patient population, including their age, sex, body weight, height, body mass index, comorbidities, diagnosis, infection origin, and surgery method (open or laparoscopic) were collected. Parameters reflecting their initial status such as the Acute Physiology and Chronic Health Evaluation II (APACHE II), American Society of Anesthesiologists (ASA), Quick SOFA (qSOFA), and SOFA scores, systemic inflammatory response syndrome (SIRS), initial systolic blood pressure (SBP), respiration rate, mental status, the presence of preoperative shock, and use of vasopressors at ED were obtained. Quick Sequential Organ Failure Assessment and SIRS were defined as positive when they fulfilled two requisite criteria. Initial postoperative parameters of the population and data regarding postoperative complications, length of treatment, and mortality rates were also collected.

There were many causes resulted the patients to have cIAI. These causes were mentioned under “Diagnosis” category (Table 1). Patients with hollow viscus perforation developed by mechanical obstruction, strangulation, and malignant neoplasm were classified as “Mechanical”. Small bowel infarction with vascular compromise also caused cIAI in many patients in our population and were classified as “Vascular”. “Ulceration” included cIAI caused by bowel perforation caused by Crohn’s disease, ulcerative colitis, and peptic ulcer disease. “Infection” category included intra-abdominal abscess formed by variety of causes, such as complicated diverticulitis, and Fournier gangrene extending to abdominal cavity.

**Table 1:** Baseline characteristics of the study population

	Culture positive (*n* = 39)	Culture negative (*n* = 200)	*P* value
Age, years	76.00 [64.00, 84.00]	70.00 [57.25, 78.00]	.031^a^
Sex, M/F, *n* (%)	18 (46.2)/21 (53.8)	116 (58.0)/84 (42.0)	.173
Body weight, kg	60.00 [51.00, 65.00]	60.00 [51.00, 66.70]	.857^a^
Height, cm	160.00 [155.00, 170.00]	163.00 [156.00, 170.00]	.603^a^
BMI, kg/m^2^	21.47 [20.28, 24.95]	22.37 [20.32, 24.22]	.989^a^
CCI, *n*	4.00 [3.00, 5.00]	4.00 [2.00, 5.00]	.846^a^
Comorbidity, *n* (%)			
HTN	20 (51.3)	101 (50.5)	.900
CAOD	2 (5.1)	19 (9.5)	.542^b^
DM	9 (23.1)	45 (22.5)	.937
CRF	1 (2.6)	11 (5.5)	1.000^b^
Malignancy	6 (15.4)	34 (17.0)	.939
COPD	3 (7.7)	8 (4.0)	.394^b^
LC	1 (2.6)	10 (5.0)	1.000^b^
Diagnosis, *n* (%)			.023
Mechanical	8 (20.5)	76 (38.0)	
Vascular	7 (17.9)	34 (17.0)	
Ulceration	11 (28.2)	61 (30.5)	
Infection	13 (33.3)	29 (14.5)	
Infection origin, *n* (%)			.012^b^
Stomach	5 (12.8)	44 (22.0)	
Duodenum	1 (2.6)	12 (6.0)	
Small bowel	7 (17.9)	68 (34.0)	
Colon	26 (66.7)	70 (35.0)	
Multifocal	0 (0.0)	6 (3.0)	
Colon involvement, *n* (%)	26 (66.7)	70 (35.0)	< .001
Perforation, *n* (%)	34 (87.2)	154 (77.0)	.156
Laparoscopy/open, *n* (%)	2 (5.1)/37 (94.9)	35 (17.5)/165 (82.5)	.054^b^

*M/F* male/female; *BMI* body mass index; *CCI* Charlson Comorbidity Index; *HTN* hypertension; *CAOD* coronary artery occlusive disease; *DM* diabetes mellitus; *CRF* chronic renal failure; *COPD* chronic obstructive pulmonary disease; *LC* liver cirrhosis

^a^Mann–Whitney *U* test

^b^Fisher’s exact test

Pulmonary consolidation and pleural effusion were recorded according to the official reports from the chest radiology specialists. Pre-existing pulmonary consolidations were excluded and those newly developed during the postoperative care were included for analysis. Infectious complications were also reviewed which is defined as newly occurred infections except for pulmonary consolidations during postoperative care based on the consensus conference definitions of infection in the ICU [21].

This study was approved by the Ajou University Institutional Review Board (AJIRB-MED-MDB-19-314), and informed consent was waived due to the retrospective design of the study.

### Statistical analysis

For all variables, data normality was tested using the Shapiro–Wilk test. Continuous variables, presented as means ± standard deviations or medians [interquartile range] depending on the data normality, were compared using the Student *t*-test or the Mann–Whitney *U* test, as appropriate. Categorical variables, presented as percentages, were evaluated with chi-squared test or Fisher’s exact test. To evaluate risk factors for positive cultures, multivariate logistic regression analysis with maximum likelihood method and backward stepwise selection was performed. Variables for multivariate logistic regression analysis included variables with *P* value < 0.05 in univariate analysis. Patient data with any missing variables were excluded from the analysis. The findings were considered statistically significant at *P* values < 0.05. Statistical analysis was performed using SPSS^®^ Statistics 25.0 (IBM Corp., Armonk, NY).

## Results

### Baseline characteristics of patients

A total of 200 patients were included in the culture-negative group and 39 patients in the culture-positive group. The baseline characteristics between the two groups were not significantly different except for their age (76.00 [64.00, 84.00] vs 70.00 [57.25, 78.00]; *P* = 0.031), their diagnoses that caused cIAI (*P* = 0.023), and their infection origins (*P* = 0.012). Among infection origins, patients in the culture-positive group had a significantly higher rate of colon involvement (66.7% vs 35.0%; *P* < 0.001) (Table 1).

### Variables associated with higher initial severity of illness

According to the ASA score (*P* = 0.041), SOFA score (7.54 ± 4.29 vs 4.55 ± 3.33; *P* < 0.001), SIRS (79.5% vs 59.0%; *P* = 0.016), SBP (101.03 ± 23.29 vs. 114.48 ± 29.95 mmHg; *P* = 0.003), respiration rate (19.87 ± 6.37 vs. 17.49 ± 4.95 per minute; *P* = 0.009), and proportion of patients who used vasopressors in ED (41.0% vs. 15.5%; *P* < 0.001), the patients in the culture-positive group showed higher severity than those in the culture-negative group (Table 2).

**Table 2:** Parameters reflecting initial severity of the illness

	Culture positive (*n* = 39)	Culture negative (*n* = 200)	*P* value
APACHE II, *n*	12.00 [9.00, 15.00]	13.00 [10.00, 18.00]	.444^a^
ASA, *n* (%)			.041^b^
1	7 (17.9)	49 (24.5)	
2	23 (59.0)	103 (51.5)	
3	7 (17.9)	48 (24.0)	
4	2 (5.1)	0 (0)	
qSOFA, *n* (%)	9 (23.1)	25 (12.5)	.084
SOFA score, *n*	7.54 ± 4.29	4.55 ± 3.33	< .001
SIRS, *n* (%)	31 (79.5)	118 (59.0)	.016
SBP, mmHg	100.00 [85.00, 110.00]	110.00 [94.00, 138.00]	.005^a^
Respiration rate, f/min	18.00 [16.00, 22.00]	16.00 [14.00, 20.00]	.004^a^
Altered mental status, *n* (%)	6 (15.4)	17 (8.5)	.182
Preoperative shock, *n* (%)	21 (53.8)	81 (40.5)	.123
ED vasopressor use, *n* (%)	16 (41.0)	31 (15.5)	< .001
Septic shock, *n* (%)	12 (30.8)	42 (21.0)	.182
IV hydrocortisone use, *n* (%)	8 (20.5)	22 (11.0)	.101

*APACHE* acute physiology and chronic health evaluation; *ASA* American Society of Anesthesiology; *SOFA* sequential organ failure assessment; *qSOFA* quick SOFA; *SIRS* systemic inflammatory response syndrome; *SBP* systolic blood pressure; *ED* emergency department

^a^Mann–Whitney *U* test

^b^Fisher’s exact test

### Postoperative complications, length of treatment, and mortality rates

More patients in the culture-positive group developed pleural effusion (89.7% vs. 72.5%; *P* = 0.025) and needed percutaneous drainage (35.9% vs. 17.5%; *P* = 0.009). The survived patients in the culture-positive group stayed in the hospital (28.00 [21.50, 34.50] vs 14.00 [10.00, 23.00] days; *P* =  < 0.001) and ICU longer (9.00 [4.00, 23.00] vs 2.00 [1.00, 6.00] days; *P* = 0.001). Patients in the culture-positive group needed more days to wean from vasopressors (3.00 [2.00, 7.00] vs 1.00 [0.00, 3.00] days; *P* < 0.001) and mechanical ventilators (4.00 [1.00, 18.00] vs 1.00 [0.00, 3.75] days; *P* < 0.001) than those in the culture-negative group.

In-hospital (35.9% vs. 14.5%; *P* = 0.001) and 30-day mortality rates (30.8% vs. 12.0%; *P* = 0.003) were also higher in the culture-positive group than the culture-negative group (Table 3). 30-day mortality rates were also higher in the patients who were infected with micro-organisms with wide range of resistance to antibiotics, such as MRSA, VRE, ESBL-producing Enterobacteriaceae, and CRE (38.5% vs 13.7%; *P* = 0.031).

**Table 3:** Postoperative complications, length of treatment, and mortality rates

	Culture positive (*n* = 39)	Culture negative (*n* = 200)	*P* value
Pleural effusion, *n* (%)	35 (89.7)	145 (72.5)	.025
PCD, *n* (%)	14 (35.9)	35 (17.5)	.009
Newly developed pulmonary consolidation, *n* (%)	12 (30.8)	36 (18.0)	.069
Reintubation, *n* (%)	4 (10.3)	24 (12.0)	1.000^a^
Infectious complications, *n* (%)	1 (2.6)	12 (6.0)	.700^a^
HLOS, days	28.00 [21.50, 34.50]	14.00 [10.00, 23.00]	< .001^b^
ICU LOS, days	9.00 [4.00, 23.00]	2.00 [1.00, 6.00]	.001^b^
Duration of antibiotics use, days	11.00 [1.00, 26.00]	8.00 [1.00, 15.75]	.073^b^
Duration of vasopressor use, days	3.00 [2.00, 7.00]	1.00 [0.00, 3.00]	.001^b^
MV day, days	4.00 [1.00, 18.00]	1.00 [0.00, 3.75]	< .001^b^
In-hospital mortality, *n* (%)	14 (35.9)	29 (14.5)	.001
30-day mortality, *n* (%)	12 (30.8)	24 (12.0)	.003

*PCD* percutaneous catheter drainage; *HLOS* hospital length of stay; *ICU LOS* intensive care unit length of stay; *MV* mechanical ventilator

^a^Fisher’s exact test

^b^Mann-Whitney *U* test

### Multivariate logistic regression analysis

Multivariate logistic regression analysis revealed that involvement of the colon (Odds ratio [OR]: 4.211; 95% confidence interval [CI] 1.909–9.287; *P* < 0.001) and higher SOFA score (OR 1.169; 95% CI 1.065–1.282; *P* = 0.001) were associated with a higher risk of positive culture (Table 4). Univariate and multivariate analysis to investigate association between patient characteristics and 30-day mortality were also performed. According to the analysis, Colon involvement (OR 3.989; 95% CI 1.595–9.975; *P* = 0.003), higher SOFA score (OR 1.305; 95% CI 1.177–1.446; *P* < 0.001), and lower SBP (OR 0.976; 95% CI 0.959–0.993; *P* = 0.007) were independently associated with 30-day mortality (Supplementary Table 1).

**Table 4:** Univariate and multivariate logistic regression analysis of baseline characteristics and initial status of the study population for culture-positivity

Variables	Univariate analysis	Multivariate analysis
Age, *n*	.031	
Colon involvement	< .001	OR 4.211, CI 1.909–9.287; *P* < .001
ASA	.041	
SOFA score, *n*	< .001	OR 1.169, CI 1.065–1.282; *P* = .001
SIRS	.016	
SBP, mmHg	.005	
Respiration rate, *n*/min	.004	
ED vasopressors use	< .001	

*ASA* American Society of Anesthesiology, *SOFA* sequential organ failure assessment, *SIRS* systemic inflammatory response syndrome, *SBP* systolic blood pressure, *ED* emergency department, *OR* odds ratio, *CI*confidence interval

### Microorganisms identified in cultures

Gram-positive cocci were the most common microorganism identified in both blood and peritoneal cultures (42.3 and 26.3%, respectively) followed by *Escherichia coli* (19.2 and 31.6%, respectively). Other microorganisms identified in the cultures were microorganisms from the *Klebsiella* genus and *Candida* species. Anaerobes, gram-positive and gram-negative rods were also identified from the cultures (Table 5).

**Table 5:** Microorganisms identified in initial blood and peritoneal cultures

Microorganism	Frequency
From blood cultures, *n* (%)	
Gram-positive cocci	11 (42.3)
* Staphylococcus aureus*	3
* Staphylococcus hominis*	2
* Staphylococcus epidermidis*	2
* Micrococcus* genus	1
* Enterococcus* genus	1
* Streptococcus anginosus*	1
* Streptococcus gordonii*	1
* Bacillus* genus	3 (11.5)
* Escherichia coli*	5 (19.2)
* Klebsiella* genus	2 (7.7)
Others	5 (19.2)
* Clostridium* genus	1
* Bacteroides* genus	1
* Fusobacterium mortiferum*	1
* Lactobacillus* genus	1
* Prevotella oralis*	1
From peritoneal cultures, *n* (%)	
Gram-positive cocci	5 (26.3)
* Staphylococcus aureus*	2
* Enterococcus faecalis*	3
* Escherichia coli*	6 (31.6)
* Klebsiella* genus	4 (21.1)
* Enterobacter* genus	2 (10.5)
* Candida* species	2 (10.5)

### Bacterial resistance to antibiotics

Resistance to methicillin in gram-positive bacteria was observed in 25.0 and 40.0% in blood and peritoneal cultures, respectively. Furthermore, half of methicillin resistant bacteria also had resistance to vancomycin. Among gram-negative bacteria from blood cultures and peritoneal cultures, 28.6 and 50.0% were ESBL-producing Enterobacteriaceae, respectively. One of them was resistant to carbapenem (Table 6).

**Table 6:** Bacterial resistance to antibiotics

	Frequency of resistance
Positive blood culture, *n* (%)	
Gram-positive bacteria	
Resistance to methicillin	4/16 (25.0)
Resistance to vancomycin	1/4 (25.0)
Gram-negative bacteria	
ESBL-producing Enterobacteriaceae	2/7 (28.6)
CRE	0/2 (0.0)
Positive peritoneal culture, *n* (%)	
Gram-positive bacteria	
Resistance to methicillin	2/5 (40.0)
Resistance to vancomycin	2/2 (100.0)
Gram-negative bacteria	
ESBL-producing Enterobacteriaceae	6/12 (50.0)
CRE	1/6 (16.7)

*ESBL* extended spectrum beta-lactamase; *CRE* carbapenem-resistant Enterobacteriaceae

## Discussion

Our study results showed that the culture-positive group had worse outcomes than the culture-negative group in terms of mortality, length of hospital and ICU stay, and mechanical ventilator days. Colon involvement and high SOFA score were independently associated with culture positivity and 30-day mortality. Bacteria identified from culture-positive patients had higher rates of resistance to commonly used antibiotics.

In our cohort of septic patients with cIAI, 83.7% of patients with sepsis were culture-negative, which was within the range of results from previous reports [9–14]. However, 83.7% is a high number among these studies. Some of those studies only included patients with severe sepsis or septic shock, but our study included patients with not only septic shock but also sepsis. Previous studies reported that the most common site of infection in culture-negative sepsis was the respiratory system [9, 13–15]. Unlike previous studies, which included patients with sepsis of various origins, our study included patients with sepsis originating from the GI tract only, and this makes our study unique.

The primary aims of this study were to evaluate the association between culture results and mortality rates of the cIAI patients. Culture-positive patients showed higher in-hospital mortality and 30-day mortality. Although some studies reported that survival rate was similar in culture-negative and culture-positive patients [9, 18] and even higher mortality rate in culture-negative severe sepsis patients [13], our study showed lower in-hospital and 30-day mortality rates in culture-negative patients. Different results were likely to be derived from different population with different range of sepsis severity and source of infection. In our cohort, the patients had a definite origin of infection to start with, which is intra-abdominal. They all had opportunities to undergo source control, as a key treatment modality. Using antibiotics was complementary only to the source control. However, in other studies with non-surgical patients or those with heterogeneous infection source, identification of target microorganisms would be a key to sepsis treatment. Culture-negativity may as well reflect being either false-negative or viral infection. In these cases, not exactly knowing what to target may delay selection of appropriate treatment, which may increase mortality rates.

The secondary aims of this study were to investigate the predictors for culture-positive sepsis in cIAI and the microorganisms in culture-positive sepsis with their resistance to antibiotics. Multivariate logistic regression analysis revealed that colon involvement and higher SOFA score were independently associated with a higher risk of positive culture. In a study conducted by Phua et al. [15], culture-positive patients had a higher portion of liver abscess as an intra-abdominal cause of infection. In our study, involvement of the colon resulted in higher risk of culture positivity and 30-day mortality regardless of their initial severity of illness (Supplementary Table 2), possibly because bacterial loads are greater in the colon than in other parts of the GI tract. Although vasopressor use in the ED and lower SBP were noted in the culture-positive group with statistical significance, they were not shown to be risk factors for culture positivity in multivariate logistic regression analysis. However, low SBP was found to be an independent predictor for 30-day mortality.

Gram-positive cocci were the most common microorganisms identified in both blood and peritoneal cultures (44.44 and 33.33%) followed by *E. coli* (18.52 and 23.81%). Other microorganisms identified in the cultures were from the *Klebsiella* genus and *Candida* species. Anaerobes, gram-positive and gram-negative rods were also identified from the cultures. In previous studies, similar results were shown in which gram-positive cocci, *E. coli*, and *Klebsiella* were the most common [11, 14, 15, 22].

Traditionally, a combination of third-generation cephalosporin and metronidazole had been used as first-line antibiotic regimen for intra-abdominal infection in our center, which is within the guidelines of the World Society of Emergency Surgery [20]. The rate of cephalosporin resistance (35.9%) in our study was more than double the rate reported by Nannan Panday et al. [9], who observed ceftriaxone resistance in 12.9% of their culture-positive patients. However, another study from a university-affiliated hospital [23] reported that 39.5% of patients with cIAI, primarily treated with a combination of ceftriaxone plus metronidazole regimen, needed to have other intravenous antibiotics added or have their regimen changed. Therefore, we suggest using broad-spectrum antibiotics with coverage of these resistant pathogens, especially for those with high SOFA scores and a high suspicion of colon involvement.

The limitations of this study include the relatively small number of culture-positive patients compared with a modest total number of patients from a single surgical ICU in a tertiary medical center. Furthermore, the cause-effect relationship is not clear between the variables since this is a retrospective study. As shown in Table 6, prevalence of the multi-drug resistant pathogens is remarkably high. Although the patients transferred to the ED after previous admission to other hospitals were excluded, some of the included patients still could have received antibiotics via any route in local clinics since they were mostly old-aged and had comorbidities.

However, there were few attempts to conduct a study to identify predictors for culture positivity in patients with cIAI-originated sepsis who underwent emergency surgery within 24 h of their ED arrival. Thus, this study could contribute to the understanding of the characteristics of sepsis originating from cIAI, which is different from the results of studies conducted in patients with sepsis in medical ICUs. More studies in this subject on a larger number of patients with prospectively collected data are necessary to clarify our findings.

To summarize, in our cohort with identifiable intra-abdominal infection who underwent emergency surgery for source control, culture-positive patients showed higher mortality rates. They also showed longer length of hospital stay, ICU stay, and mechanical ventilator use. The patients with colon involvement were 4.2 times more likely to be culture-positive, and as SOFA score increased 1 point, the risk of culture-positivity increased by 1.2 times. We suggest using antibiotics susceptible to pathogens with wide range of resistance in a selected group of patients with high risk of mortality who have colon involvement and higher SOFA score, which could be translated to a high risk of culture positivity and antibiotic resistance.

## Electronic supplementary material

Below is the link to the electronic supplementary material.

Supplementary file1 (DOCX 21 kb)

Supplementary file1 (DOCX 17 kb)
