# Supplementary material for: Prediction of culture-positive sepsis and selection of empiric antibiotics in critically ill patients with complicated intra-abdominal infections: a retrospective study
Source: Eur J Trauma Emerg Surg. 2020 Nov 3;48(2):963–71. doi: 10.1007/s00068-020-01535-6 (PMC7609359; doi:10.1007/s00068-020-01535-6)
Supplement: Supplementary file 1 — Supplementary file1 (DOCX 21 kb) [file 68_2020_1535_MOESM1_ESM.docx]

Supplementary table 2. Parameters reflecting initial status of the patients according to colon involvement

|  | Colon involvement present  (n= 96) | Colon involvement absent  (n=143) | *P* value |
| --- | --- | --- | --- |
| APACHE II, n | 11.00 [9.00, 17.00] | 13.00 [10.00, 18.00] | 0.154^a^ |
| ASA, n(%) |  |  | 0.015^b^ |
| 1 | 29 (30.2) | 27 (18.9) |  |
| 2 | 50 (52.1) | 76 (53.1) |  |
| 3 | 15 (15.6) | 40 (28.0) |  |
| 4 | 2 (2.1) | 0 (0.0) |  |
| qSOFA, n(%) | 14 (14.6) | 20 (14.0) | 0.897 |
| SOFA score, n | 4.00 [2.00, 7.00] | 4.00 [2.00, 6.00] | 0.941^a^ |
| SIRS, n(%) | 27 (28.1) | 32 (22.4) | 0.616 |
| SBP, mmHg | 105.00 [90.00, 126.00] | 110.00 [91.00, 130.00] | 0.590^a^ |
| Respiration rate, f/min | 16.00 [14.00, 20.00] | 16.00 [14.00, 20.00] | 0.929^a^ |
| Altered mental status, n(%) | 11 (11.5) | 12 (8.4) | 0.431 |
| Preoperative shock, n(%) | 44 (45.8) | 58 (40.6) | 0.419 |
| ED vasopressor use, n(%) | 22 (22.9) | 25 (17.5) | 0.300 |
| Septic shock, n(%) | 23 (24.0) | 31 (21.7) | 0.679 |
| IV hydrocortisone use, n(%) | 96 (9.4) | 21 (14.7) | 0.224 |

Values are presented as means ± standard deviations, medians [interquartile range], or n (%).

APACHE, acute physiology and chronic health evaluation; ASA, American Society of Anesthesiology; SOFA, sequential organ failure assessment; qSOFA, quick SOFA; SIRS, systemic inflammatory response syndrome; SBP, systolic blood pressure; ED, emergency department.

^a^Mann-Whitney U test.

^b^Fisher’s exact test
