# Supplementary material for: Prediction of culture-positive sepsis and selection of empiric antibiotics in critically ill patients with complicated intra-abdominal infections: a retrospective study
Source: Eur J Trauma Emerg Surg. 2020 Nov 3;48(2):963–71. doi: 10.1007/s00068-020-01535-6 (PMC7609359; doi:10.1007/s00068-020-01535-6)
Supplement: Supplementary file 2 — Supplementary file1 (DOCX 17 kb) [file 68_2020_1535_MOESM2_ESM.docx]

Supplementary table 1. Univariate and multivariate analysis of association between patient characteristics and factors predictive of 30-day mortality

| Variables | Univariate analysis | Multivariate analysis |
| --- | --- | --- |
| Colon involvement | .016 | OR 3.989, CI 1.595-9.975; *P* =.003 |
| ASA | .044 |  |
| qSOFA | <.001 |  |
| SOFA score | <.001 | OR 1.305, CI 1.177-1.446; *P* < .001 |
| SIRS | .005 |  |
| SBP | <.001 | OR 0.976, CI 0.959-0.993; *P* = .007 |
| Respiration rate | .013 |  |
| AMS | .001 |  |
| ED vasopressor use | <.001 |  |
| Septic shock | <.001 |  |
| Culture positivity | 0.003 |  |

ASA, American Society of Anesthesiology; SOFA, sequential organ failure assessment; qSOFA, quick SOFA; SIRS, systemic inflammatory response syndrome; SBP, systolic blood pressure; AMS, altered mental status; ED, emergency department; OR, odds ratio; CI, confidence interval.
